# Melanocortin-4 Receptor and Lipocalin 2 Gene Variants in Spanish Children with Abdominal Obesity: Effects on BMI-SDS after a Lifestyle Intervention

**DOI:** 10.3390/nu11050960

**Published:** 2019-04-26

**Authors:** Lydia Morell-Azanza, Ana Ojeda-Rodríguez, Johanna Giuranna, Mª Cristina Azcona-SanJulián, Johannes Hebebrand, Amelia Marti, Anke Hinney

**Affiliations:** 1 Department of Nutrition, Food Sciences and Physiology, University of Navarra, c/Irunlarrea, 1. 31008 Pamplona, Spain; lmorell.1@alumni.unav.es (L.M.-A.); aojeda.5@alumni.unav.es (A.O.-R.); 2 IdiSNA, Instituto de Investigación Sanitaria de Navarra, c/Irunlarrea, 3. 31008 Pamplona, Spain; cazcona@unav.es; 3 Department of Child and Adolescent Psychiatry, Psychosomatics and Psychotherapy, University Hospital Essen, University of Duisburg-Essen, Virchowstr. 174, D-45147 Essen, Germany; johanna.giuranna@uni-due.de (J.G.); johannes.hebebrand@uni-duisburg-essen.de (J.H.); anke.hinney@uni-due.de (A.H.); 4 Department of Paediatrics, Clínica Universidad de Navarra, Paediatric Endocrinology Unit, c/Piío XII, 36. 31008 Pamplona, Spain; 5 Biomedical Research Centre Network on Obesity and Nutrition (CIBERobn), Physiopathology of Obesity and Nutrition, Institute of Health Carlos III, Av. Monforte de Lemos, 3-5. 28029 Madrid, Spain

**Keywords:** childhood obesity, CEBQ, eating behavior and Ile251Leu

## Abstract

Mutations leading to a reduced function of the melanocortin-4 receptor (MC4R) exert a major gene effect on extreme obesity. Recently it was shown that the bone derived hormone lipocalin 2 (LCN2) binds to the MC4R and activates a MC4R dependent anorexigenic pathway. We identified mutations in both genes and screened the effects of *MC4R* and *LCN2* mutations on eating behavior and weight change after a lifestyle intervention. One hundred and twelve children (11.24 ± 2.6 years, BMI-SDS 2.91 ± 1.07) with abdominal obesity participated in a lifestyle intervention. *MC4R* and *LCN2* coding regions were screened by Sanger sequencing. Eating behavior was assessed at baseline with the Children Eating Behavior Questionnaire (CEBQ). We detected three previously described non-synonymous *MC4R* variants (Glu42Lys, Thr150Ile, and Arg305Gln) and one non-synonymous polymorphism (Ile251Leu). Regarding *LCN2*, one known non-synonymous variant (Thr124Met) was detected. Eating behavior was described in carriers of the *MC4R* and *LCN2* mutation and in non-carriers. *MC4R* and *LCN2* mutations were detected in 2.42% and 0.84%, respectively, of Spanish children with abdominal obesity. A number of subjects with functional mutation variants in *MC4R* and *LCN2* were able to achieve a reduction in BMI-SDS after a lifestyle intervention.

## 1. Introduction

Obesity has been defined by the World Health Organization (WHO) as an abnormal or excessive body fat accumulation that may impair health [1]. Both environmental and genetic factors have an influence on weight gain [2]. The impact of genetics on obesity is heterogeneous. For a small number of subjects obesity is caused by mutations in single genes, for the majority of the population obesity has a polygenic nature [2,3]. 

One of the most common single genes harboring variants associated with obesity is the melanocortin-4 receptor gene (*MC4R*) [4,5]. It is known as a regulator of energy homeostasis due to its effect on food intake and energy expenditure via neuronal melanocortinergic pathways [6]. More than 369 mutations including non-synonymous, nonsense, and frameshift mutations have been identified mainly in obese individuals [7,8]. Most of the non-synonymous mutations lead to partial or complete loss of function of the *MC4R* [9]. The polymorphism Ile251Leu leads to an increased function of MC4R and is associated with a reduced BMI [7]. 

The protein lipocalin 2 has been associated with obesity [10,11] and was thought to be secreted from adipose tissue as an adipokine [12]. However recent studies discovered a ten-fold higher expression in osteoblasts than in adipose tissue. In this line, recent studies have shown that lipocalin 2 (LCN2) binds to the MC4R to activate MC4R-dependent anorexigenic pathways [13]. It has also been observed that obese participants heterozygous for *MC4R* mutations leading to impaired function have higher levels of plasma LCN2 than BMI-matched MC4R wild-type controls [13].

Our aim was to screen for mutations in the *MC4R* and *LCN2* genes in a Spanish pediatric population with abdominal obesity. Moreover, we investigated the effects on eating behavior and weight change after participating in a one-year lifestyle intervention for carriers versus non-carriers of *MC4R* and *LCN2* variants.

## 2. Materials and Methods 

### 2.1. Subjects

For this study, a total of 112 children between 7 to 16 years of age and with abdominal obesity defined as a waist circumference higher than the 90th percentile [14] participate in a lifestyle intervention. The IGENOI study is a 2-year lifestyle intervention program for children with abdominal obesity carried out by GENOI group *(“Grupo de Estudio Navarro de la Obesidad infantil”*). This study is a randomized control trial (NCT03147261) conducted in Pamplona, Spain. Children were recruited from the Pediatric Endocrinology at “Clinica Universidad de Navarra” and “Complejo hospitalario de Navarra”, and from health care centers around Pamplona and its neighborhoods. 

Participants with previous diabetes, presence of other diseases beside obesity, major psychiatric illness including bulimia nervosa, pharmacological treatment, food intolerance, or treatment with special diets, or frequent alcohol or drug consumption were excluded. Children and their parents signed a written informed consent in the screening visit. The study protocol was performed in accordance with the ethical standards of the Declaration of Helsinki (Fortaleza, Brasil, October 2013), and was approved by the ethics committee of the University of Navarra (Reference number 044/2014).

### 2.2. Experimental Design

The lifestyle intervention comprises an 8-week intensive phase (*N* = 104 subjects) and a follow-up period of 10 months (*N* = 85 subjects, 1 year). The dropout rate was 7% at week 8 and 24% at 1 year of follow up.

A multidisciplinary team conformed by dietitians, pediatricians, nurses, physical activity experts, and laboratory technicians were involved in the development of the study protocol. Participants were randomly assigned into two different groups: intensive care and usual care group, following a 3:1 ratio. The first one was given a moderate hypocaloric Mediterranean diet, as previously described [15], while the usual care group received usual pediatric advice with healthy diet recommendations [16]. Specifically, intensive care participants were prescribed with a moderate hypocaloric diet based on a fixed full-day meal plan. The restriction, not to interfere with growth, was calculated depending on the degree of obesity (from −10% to −40% of total energy intake). A Mediterranean-style diet based consists of high consumption of fruit, vegetables, whole grains, legumes, nuts, seeds, and olive oil, minimally processed foods; moderate consumption of dairy products, fish, and poultry; and low consumption of red meat [15]. Both groups were encouraged to increase their physical activity to at least 200 minutes per week as recommended by The American College of Sports Medicine to prevent weight gain [17]. 

The intervention group participants had 30-minute individual sessions with the dietitian every two weeks during the 8-week treatment phase. In these sessions a follow-up about the accomplishment of the diet and anthropometric measurements was performed. In addition, the parents or legal guardians had one parallel group session where they received education about their role in the study and obesity related problems, while intensive care participants were encouraged to make healthy lifestyle decisions about food choices, eating behavior, sedentary activities, and physical activity. On the other hand, usual care participants and their parents received one 30-min individual session with the dietitian and five monitoring visits to assess anthropometric parameters. 

During the follow-up period, participants had monitoring visits at 3, 4,5,6,9, and 12 months from the baseline visit. 

### 2.3. Anthropometric, Clinical and Biochemical Measurements

All anthropometric measurements were performed at baseline, after 8 weeks, and at 1 year of follow up according to standard procedures and calibrated tools in the paediatric population [18]. Measurements were performed by trained personnel in a wide space, while the participants were asked to stand barefoot and wore light clothing, without hair ornaments or jewels. 

Body weight and body fat were determined using a digital scale BC-418 Segmental Body Composition Analyzer according to manufacturer instructions (Tanita, Tokyo, Japan). To measure height, participants were asked to stand on stadiometer with the feet placed parallel and slightly apart, and heels, buttocks, scapula, and occipital head area touching the vertical board at the same time. A non-stretchable measuring tape (type SECA 200) was used for measuring waist circumference (WC) and hip circumference (HC) by standard procedures (Type SECA 200). The waist-to-hip and waist-to-height ratios were also calculated. 

Body mass index was calculated as weight divided by squared height (Kg/m^2^). BMI-standard deviation (BMI-SDS) is BMI values converted into standard deviation using age and specific cut-points according to Spanish reference growth charts [19]. Pubertal status was determined using Tanner stage and was evaluated by a paediatrician. The presence of *Acanthosis Nigricans* was diagnosed by pediatricians of the team at baseline. Blood pressure was measured following standard procedures, as described elsewhere [20]. 

Venous blood samples were obtained by specialized trained nurses at the Hospital after an overnight fast. Glucose, insulin leptin, and lipid profiles were determined by standard autoanalyzer techniques at baseline. Insulin resistance was calculated from the homeostasis model assessment of insulin resistance (HOMA-IR). 

### 2.4. Physical Activity

Moderate-to-vigorous physical activity was assessed at baseline using triaxial accelerometry (Actigraph wGT3X-BT, Actigraph LLC, Penascola, FL, USA). Briefly, participants wore the accelerometer around the non-dominant waist for four days, including, at least, two weekend days, as described elsewhere [21]. Accelerometry data were analysed using ActiLife 6.0 software (Actigraph LLC, Penascola, FL, USA) as describe elsewhere [22]. 

### 2.5. Children Eating Behavior Questionnaire (CEBQ)

The Children Eating Behaviour Questionnaire (CEBQ) is a multidimensional parent-reported questionnaire about their children’s eating behaviour [23]. It includes 35 items regarding eating styles that are clustered into eight subscales. The eight subscales are classified into two dimensions: food approach or food avoidance. The food approach dimension comprises the following subscales: “Emotional overeating”, “Enjoyment of food”, “Desire for drink”, and “Food responsiveness”. Food avoidance is represented by the following: “Slowness in eating”, “Satiety responsiveness”, “and Emotional undereating”, and “Food fussiness”. It was fulfilled by parents or legal guardians at baseline. 

For each behaviour subscale parents report their children’s behaviour on a five-point Likert Scale, that ranges from never, rarely, sometimes, often, or always (1 to 5). A ratio was calculated between the sums of the food approach vs. the sum of the food avoidance subscales.

### 2.6. DNA Extraction

Venous blood samples were obtained on ethylendiaminetetraacetic acid (EDTA) tubes, which were centrifuged 30 minutes after the extraction at 3500 rpm for 15 minutes at 4 °C. DNA was extracted from the buffy coat fraction using a commercial kit MasterPure DNA purification kit for Blood Version II (Epicenter Biotechnologies, Madison, WI, USA). Its quality and quantity were determined with a Nanodrop spectrometer ND-1000 (Nanodrop Technologies, Wilmington, Delware, USA) and it was stored at −80 °C until processing.

### 2.7. Mutation Screen

*MC4R* and *LCN2* genes were screened for mutations. All subjects were heterozygous for the mutations. *MC4R* was analyzed with one PCR fragment, while *LCN2* was divided into four PCR fragments. Methodological details can be obtained from the authors.

All samples were commercially Sanger sequenced by LGC Genomics (Berlin, Germany). Analyses of the sequences were performed by two experienced individuals in Essen (Germany). Samples with discrepancies were re-sequenced by Seqlab laboratories (Göttingen, Germany).

### 2.8. Statistical Analysis

Statistical analyses were performed using STATA version 12 (StataCorp, College Station, TX, USA). Normality was assessed by Shapiro-Wilk. All the tests were two-sided and the significance level was set at α = 0.05. We did not correct for multiple testing.

We used Wilcoxon rank-sum test for the comparison between subjects with Ile 251Leu MC4R mutation and without MC4R mutation for the variables studied at baseline. 

Paired t-test was applied for assessing changes in BMI-SDS between baseline vs. week 8, and baseline vs. 1 year. Furthermore, we performed a comparison between subjects with Ile 251Leu MC4R mutation and without MC4R mutation for changes in BMI-SDS (week 8 and one year, Wilcoxon rank-sum test).

## 3. Results

One hundred and twelve children with abdominal obesity (mean age 11.24 years, males 38%, BMI-SDS 2.93) participated in the study.

### 3.1. Description of Identified Variants 

Mutation screen of the coding region of MC4R identified a total of four variants, three known nonsynonymous variants, rs776051881 (Glu42Lys), rs766665118 (Thr150Ile), and rs775382722 (Arg305Gln), and one nonsynonymous polymorphism rs52820871 (Ile251Leu) (Table 1). First, we classified variants as leading to reduced receptor function or similar to the wild type MC4R function. Three previously reported variants (Glu42Lys, Thr150Ileu, and Arg305Gln) lead to a reduced MC4R function as classified by in-silico predictors [24] that claim them to be disease causing. Furthermore, some in-vitro analyses had described reduced function for the mutations Thr150Ileu and Arg305Gln [9,25,26]. Our study shows a frequency of 2.52% of MC4R mutations leading to a reduced function in our sample of Spanish of children with abdominal obesity.

The mutation screen of the coding region of LCN2 identified a total of twelve variants, eleven are located in intronic regions (rs2232632, rs202024127, rs11794980, rs2232629, rs2232625, rs2232626, rs116745581, rs2232628, rs568419305, rs2232631, rs2232632) and one is a known nonsynonymous variant: rs79993583 (Thr124Met). The analysis of all detected variants by in-silico predictors show that three of them could be disease causing (Supplementary Table S1). 

### 3.2. Phenotypic Description of Mutation Carriers

In Table 2 phenotypic characteristics of participants with mutations at MC4R and LCN2 genes at baseline are described in detail. We also provide data from obese participants of IGENOI study without mutations in the coding regions of these genes. 

For 103 children (37.8% males) with a mean age of 11.32 years no mutations in MC4R and LCN2 genes were found, they are wild type carriers. Mutated subjects are compared with them in the subsequent analyses. All wild type carriers were also obese (BMI-SDS: 2.92 ± 1.10) and 41.7% showed clinical evidence for insulin resistance in the presence of Acanthosis Nigricans that was accompanied by a HOMA-IR of 4.03 ± 3.17. 

A 14 year-old girl was heterozygous for the Glu42Lys mutation at the MC4R gene. She suffers from severe-obesity (BMI-SDS:4.04) reflected in a fat mass of 40.8%. The main clinical features of this participant were leptin levels higher than expected for the BMI [32], and the presence of insulin resistance observed as hyperinsulinemia, increased HOMA-IR (4.23), and Acanthosis Nigricans. 

Mutation Thr150Ileu at the MC4R gene was observed in an eight year-old boy with a BMI-SDS of +3.5. Biochemical parameters were normal and the participant was physically active (50.65 min/day). One participant carries two variants at the MC4R gene: Arg305Gln and the polymorphism Ile251Leu. She is a 12 year-old girl with a BMI-SDS of 2.91, and a fat mass of 43.2% (measured by bioimpedance). Five independent children also carried the polymorphism Ile251Leu. They had a mean age of nine years and a mean BMI-SDS of 2.69. In comparison with the participants with the wild type receptor, none of the participants carrying this polymorphism showed Acanthosis Nigricans. 

The polymorphism Ile251Leu of MC4R was observed in five participants with a mean age of nine years old. We found increased levels of total cholesterol and LDL-cholesterol in comparison to participants without MC4R mutation (*p* = 0.004 and *p* = 0.013; Wilcoxon rank-sum test). These differences persist when subjects with the Ile251Leu MC4R mutation were compared to age and sex matched subjects without MC4R mutation (Supplementary Table S2). 

One male 15 year-old heterozygously carried the mutation Thr124Met at the LCN2 gene. Clinical characteristics of this participant included severe obesity (BMI-SDS = +4.01) accompanied by an elevated percentage of fat mass (38.7%). Also, a remarkable difference of higher levels of MVPA was detected in comparison with the wild type population. The carrier of this mutation was much more active than the wild-type controls (120.92 min/day vs. 44.82 min/day, *p* = 0.088). We also evaluated the siblings (three girls) of the index patient, all of them were obese. Two of them were also carriers of Thr124Met (BMI-SDS: 3.47 and BMI-SDS: 3.76). The MAF for this variant in the LCN2 gene in the study population is 0.44%.

### 3.3. Children Eating Behavior

**Figure 1:**
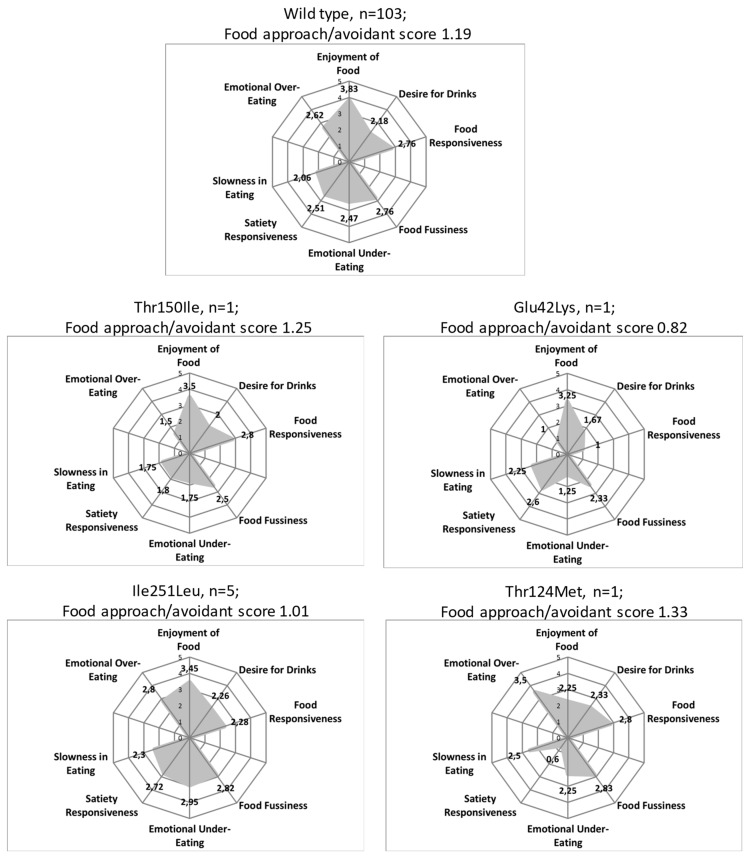
Childhood Eating Behavior Score (CEBQ) in Spanish children with abdominal obesity. The Food approach/avoidant score refers to the quotient between the sums of scores of the “food approach” subscales divided by the sum of the scores of the “food avoidant” subscales. Subjects with mutations in MC4R (Thr124Met, Thr150Ileu) and LCN2 (Thr124Met) genes were evaluated.

The eating behavior scores with four dimension charts of children with abdominal obesity at baseline is described in Figure 1. Wild type subjects (no mutations in MC4R and LCN2 genes) had a score of 1.19. Carriers of the MC4R Thr150Ileu and LCN2 Thr124Met variants had nominally higher scores than wild type subjects, while participants with Ile251Leu polymorphism have lower score. 

### 3.4. Change in BMI-SDS and Mutations in MC4R and LCN2 After 8-Week and 1-Year of Follow Up

Carriers of mutations that lead to a reduced function in MC4R and LCN2 genes showed a disparity of responses to the lifestyle intervention (Table 3). Changes in BMI-SDS of the five subject carriers of the Ile251Leu polymorphism are quite variable with two of them not showing any improvement at 1 year. Moreover, no significant differences in BMI-SDS changes were found after eight weeks or one-year of follow up in mutated carriers vs. non carriers subjects in analysis conducted in matched age and sex subjects with and without a functional mutation in MC4R (Supplementary Table S3).

## 4. Discussion

To our knowledge, this is the first study evaluating *MC4R* and *LCN2* gene variants, in a population of children and adolescents with abdominal obesity. In addition, we measured the eating behavior in all participants with the CEBQ. Finally, we reported changes in BMI-SDS achieved after eight weeks and 1 year of follow-up according to the two different strategies (usual care or intensive care) and the presence of *MC4R* and *LCN2* gene variants. 

The rate of participants carrying a *MC4R* mutation leading to a reduced function was 2.67 %. This frequency is in accordance with the reported values in Czech children with obesity being 2.4% [33]. Previously, a wide variability (ranging from 0.5 to 5.8%) in the frequency of *MC4R* mutations had been described in children and adolescents with obesity in different populations [33,34,35,36].

In our population, we found one extremely obese participant (BMI-SDS +4.04) carrying the *MC4R* mutation Glu42Lys. This mutation was previously reported in 5.6% of Turkish children with obesity [37]. In our case, the minor allele frequency (MAF) for this mutation was 0.89%.

Moreover, an eight year-old boy with a BMI-SDS of +3.5 was a heterozygous carrier of the *MC4R* Thr150Ileu variation. This mutation had previously been described in an obese Chilean child with a BMI-SDS of +2.79 [38]. In the Chilean population the MAF for this variant was 0.45% similar to the observed value in our Spanish population (0.84%). 

Mutation Arg305Gln of the *MC4R* gene was characterized in functional studies as a variation that causes a decrease in MC4R constituently activity and in the response to the agonist [9]. We found this mutation in a 12 year-old girl with obesity (43.2% body fat mass and BMI-SDS +2.91). This participant also carries the Ile251Leu polymorphism. There is also a German obese boy harboring this Arg305Gln variation (BMI-SDS +2.5) [39]. The frequency of this variation in our population is 0.84% while in the German population was 0.19%.

Two polymorphisms in the *MC4R* (Val103Ileu and Ile251Leu) had been demonstrated to reduce the risk of obesity [40,41,42]. Val103Ileu was described in white Europeans with frequencies between 1–4% [2]. In our study, we did not observe carriers of this variation. In other independent studies in Spanish children the frequencies of this polymorphism were lower than in other European countries [43,44,45]. The frequency of the Ile251Leu variation in our studied sample was 5.04% (6 children with abdominal obesity), which is higher than the observed values in other Spanish and Polish pediatric populations [28,43,44]. Heterozygous subjects for the Ile251Leu SNP showed higher levels of total cholesterol and LDL-cholesterol. There are studies on the association between *MC4R* variants and other lipid markers in several populations [31,46]. 

To our knowledge this is the first study evaluating non-common variants in the *LCN2* gene in children with abdominal obesity. We found one heterozygous carrier of the Thr124Met variant that was severely obese (BMI-SDS: + 4.01) despite being physically active (more than 2 hours per day on moderate-to-vigorous physical activity).

It has been demonstrated that LCN2 suppresses appetite by signaling trough MC4R [13]. For this reason we evaluated eating behavior in our population with the children eating behavior questionnaire (CEBQ), for which solid reproducibility and high internal consistency had been reported [47]. In particular we represent graphically the multidimensionality of participants carrying *MC4R* and *LCN2* mutations. We observed that carriers of the Thr150Ile (*MC4R*) and Thr124Met (*LCN2*) variants showed slightly higher eating behavior scores than obese individuals without *MC4R* variants. This tentatively suggests an effect of these mutations on eating behavior, as mutation carriers showed lower scores on food avoidance subscales. However, as the number of mutation carriers is very low, meaningful statistical analyses are not possible.

Regarding the mutation Thr150Ile of the *MC4R* gene, an association between this variant and eating behavior in three Chilean obese carriers was reported. These participants had a cognitive restraint measured by the TFEQ-R18 questionnaire [48]. 

The participant carrying the *LCN2* Thr124Met allele showed lower satiety responsiveness (0.6 out of 5). This could be explained by a potential effect of LCN2 on appetite-suppressing activities [13]. Participants carrying the Ile251Leu polymorphism of the *MC4R* had lower scores in satiety responsiveness than the wild type population (1.01 vs. 1.19). Nevertheless, carrying the infrequent allele at *MC4R* Ile251Leu seems not to have an influence on eating behavior compared to children with obesity and variations in the *MC4R* gene. A previous study had described association between *MC4R* polymorphism rs17782313 (in the 3’ region of the gene) and childhood eating behavior. Satiety responsiveness dimension was decreased and enjoyment of food was increased in carriers of the CC allele [38]. However, the lack of significance in our results should be seen under the limited statistical power as we analyzed rare variants in a relatively small study group. 

In our population, participants carrying mutations in *MC4R* and *LCN2* that lead to a reduced function were able to achieve similar or greater reduction in BMI-SDS than children without mutation in these genes after a lifestyle intervention. These results are in concordance with previous weight loss MC4R studies [4,39]. It had been demonstrated that children with mutations in *MC4R* had a significantly greater beneficial effect from the short lifestyle interventions than wild type carriers [49]. However, in our study one carrier of Arg305Gln only achieved a successful reduction in BMI-SDS after one year of intervention. When the polymorphism Ile251Leu of the *MC4R* gene was analyzed, some participants showed a reduction in BMI-SDS similar to that of wild type subjects after the intervention. Limitations of our study comprise of a relatively small sample size, the lack of a normal-weight study group, that no functional in vitro tests were performed, and also that we cannot exclude the presence of mutations in other genes involved in monogenic obesity. 

## 5. Conclusions

In summary, *MC4R* and *LCN2* mutations were detected in 2.42% and 0.84%, respectively, of Spanish children with abdominal obesity. Our data suggests a putative association between profiles of eating behavior and functional mutations in *MC4R* gene. Specifically, *MC4R* and *LCN2* mutation carriers having abdominal obesity were able to reduce BMI-SDS after a lifestyle intervention.

## Figures and Tables

**Table 1:** MC4R and LCN2 genetic variants in Spanish children with abdominal obesity.

Gene Subject	N° of Subjects	Aminoacid Exchange	rs Number	In-silico Prediction*	Function	Reference of Functional Analysis
MC4R						
Mutations						
Glu42Lys	1	p.Glu42Lys	rs776051881	Disease causing	Not known	-
Thr150Ile	1	p.Thr150Ile	rs766665118	Disease causing	Reduced	[9,25]
Arg305Gln	1	p.Arg305Gln	rs775382722	Disease causing	Reduced	[26]
Polymorphisms						
Ile251Leu	5	p.Ile251Leu	rs52820871	Disease causing	Like wild type	[27,28,29,30,31]
LCN2						
Mutations						
Thr124Met	1	p.Thr124Met	rs79993583	Probably harmless	-	-

Abdominal obesity was defined as waist circumference (WC) above the sex and age-specific 90th percentile. * In-silico prediction was performed by mutation taster www.mutationtaster.org.

**Table 2:** Baseline characteristics of heterozygous MC4R and LCN2 variant carriers and wild type Spanish children with abdominal obesity.

		MC4R Mutations				LCN2 Mutation
	Wt Population	Glu42Lys	Thr150Ile	Arg305Gln+Ile251Leu	Ile251Leu	Thr124Met
N	103	1	1	1	5	1
Age (years)	11.32 (2.46)	14	8	12	9 (1)	15
Sex (Male/Female)	39/64	Female	Male	Female	2/3	Male
Tanner (I/II/III/IV/V)	31/17/18/6/24	V	I	II	4/-/1/-/-	V
Height (cm)	151.23 (12.72)	168	137.8	151	137.3 (13.86)	175.9
Weight (Kg)	66.71 (18.77)	97	49.1	67.2	49.94 (17.87)	112.9
BMI (Kg/m^2^)	28.55 (4.51)	34.4	25.85	29.5	25.64 (3.53)	36.5
BMI-SDS	2.92 (1.10)	4.04	3.5	2.91	2.69 (1.05)	4.01
WHR	0.88 (0.06)	0.90	0.89	0.93	0.86 (0.04)	0.82
% fat mass	37.22 (6.33)	40.8	32.1	43.2	33.98 (9.28)	38.7
Acantosis nigricans (+/-)	43/52	+	-	-	0/5	+
Glucose (mg/dL)	89.04 (6.58)	85	88	78	88.25 (6.84)	87
Insulin (μu/mL)	17.92 (13.29)	20.2	7	13.5	11.87 (6.43)	11.4
HOMA-IR	4.03 (3.17)	4.23	1.52	2.6	2.66 (1.68)	2.44
Total Colesterol (mg/dL)	162.65 (24.97)	116	162	157	198.75 (14.88)*	160
HDL-colesterol (mg/dL)	46.70 (9.96)	42	53	46	56.75 (12.25)	41
LDL-colesterol (mg/dL)	97.67 (21.15)	64	96	97	125.75 (16.82)*	99
Triglycerides (mg/dL)	94.48 (44.73)	49	64	68	81.25 ( 41.65)	98
Leptin (ng/mL)	36.41 (18.60)	90.8	14	NA	33.52 (13.11)	8.1
MVPA (min/day)	44.88 (23.69)	37.5	50.65	30.93	44.55 (18.37)	120.92
CEBQ ratio	1.22 (0.42)	0.82	1.25	NA	1.01 (0.12)	0.85

Data are expressed as mean (SD), * *p* < 0.05 for the comparison between Ile 251Leu MC4R mutation subjects and subjects without MC4R mutation, Wilcoxon rank-sum test was applied Abdominal obesity was defined as WC above the sex and age-specific 90th percentile. BMI-SDS, standard deviation score for body mass index; CEBQ, Children Eating Behavior Questionnaire Ratio; MVPA, moderate to vigorous physical activity; WHR: waist to height ratio.

**Table 3:** Changes in BMI-SDS according to MC4R and LCN2 variants after an 8-week and 1-year lifestyle intervention.

		ΔBMI-SDS			
		8 Week		1 Year	
		Mutation Carriers	Non Carriers	Mutation Carriers	Non Carriers
Usual Care Group			*n* = 27 −0.44 (0.66)***		*n* = 22 −0.47 (0.52)***
*MC4R*: Glu42Lys		− 0.51		Drop out	
*MC4R*: Thr150Ile		−0.67		−0.90	
Intensive Care group			*n* = 68 −0.51 (0.38)***		*n* = 56 −0.60 (0.72)***
*MC4R*: Arg305Gln + Ile251Leu		−0.13		−0.81	
*MC4R*: Ile251Leu	Mean (*n* = 5)	−0.74 (0.41)**		−1.02 (1.21)	
	Carrier 1	−0.95		−1.47	
	Carrier 2	−0.10		0.07	
	Carrier 3	−0.56		0.38	
	Carrier 4	−1.14		−1.56	
	Carrier 5	−0.97		−2.53	
*LCN2:* Thr124Met		−0.59		Drop out	

Data are expressed as mean (SD). Paired t-test for changes between baseline vs. 8 week, and baseline vs. 1 year was applied (** < 0.010, *** < 0.001).

## Supplementary Materials

The following are available online at https://www.mdpi.com/2072-6643/11/5/960/s1, Table S1: *LCN2* genetic variants in Spanish children with abdominal obesity. Table S2: Baseline characteristics from a subpopulation of matched age and sex subjects with the Ile251Leu MC4R mutations and without the mutation. Table S3: Changes in anthropometric and biochemical from a subpopulation of matched age and sex subjects with the Ile251Leu MC4R mutations and without the mutation.
