# Supplementary material for: Melanocortin-4 Receptor and Lipocalin 2 Gene Variants in Spanish Children with Abdominal Obesity: Effects on BMI-SDS after a Lifestyle Intervention
Source: Nutrients. 2019 Apr 26;11(5):960. doi: 10.3390/nu11050960 (PMC6566731; doi:10.3390/nu11050960)
Supplement: Supplementary file 1 [file nutrients-11-00960-s001.pdf]

Supplementary Table 1. *LCN2* genetic variants in Spanish children with abdominal obesity

Subject	Nº of families	Location	Altered codon	rs number	*In-silico* prediction
Mutations					
	2	Intron c.375+88	-	rs2232632	Probably harmless
	1	Intron c.376-9	-	rs202024127	Disease causing
	3	Intron c.713-28	-	rs2232629	Probably harmless
	1	Intron c.510-119		rs2232625	Probably harmless
	1	Intron c.593+8	-	rs2232626	Disease causing
	1	Intron (splice región) c.593-3	-	rs116745581	Disease causing
	1	Intron c.713+50	-	rs2232628	Probably harmless
	1	Intron c.593+77		rs56841305	Probably harmless
	1	Intron c.848+69		rs2232631	Probably harmless
	1	Intron c.848+90	-	rs2232632	Probably harmless
Thr124Met	1	Exon	ACG→ATG	rs7999358	Probably harmless
Polymorphisms					
	10	Intron c.593-15		rs11794980	Probably harmless

**In-silico* prediction was performed by mutation taster.

Supplementary Table 2. Baseline characteristics from a subpopulation of matched age and sex subjects with the Ile251Leu MC4R mutations and without the mutation.

	Ile251Leu MC4R mutation (n=5)	No MC4R mutation (n=22)	P[1]
**Age**	9(1)	9.36 (0.78)	0.382
**Sex (Male/Female)**	2/3	7/15	0.726
**Tanner (I/II/III/IV/V)**	4/-/1/-/-	13/6/3/-/-	0.451
**Height (cm)**	137.38 (13.86)	143.63 (8.28)	0.191
**Weight (Kg)**	49.94 (17.87)	52.97 (9.51)	0.592
**BMI (Kg/m^2)**	25.64 (3.53)	25.49 (2.47)	0.914
**BMI-SDS**	2.69 (1.05)	2.45 (0.76)	0.556
**WHR**	0.558 (0.02)	0.552 (0.03)	0.713
**% fat mass**	33.98 (9.28)	35.71 (3.62)	0.495
Acanthosis nigricans (+/-)	5/-	13/9	0.080
**Glucose (mg/dL)**	88.25 (6.84)	86.5 (5.62)	0.593
**Insulin (μU/mL)**	11.87 (6.43)	15.29 (10.58)	0.549
**HOMA-IR**	2.66 (1.68)	3.32 (2.47)	0.620
**Total cholesterol (mg/dL)**	198.75 (14.88)	164 (23.64)	0.011
**HDL-cholesterol (mg/dL)**	56.75 (12.25)	49.11 (2.58)	0.229
**LDL-cholesterol (mg/dL)**	125.75 (16.82)	97.08 (22.31)	0.027
**Triglycerides (mg/dL)**	81.25 (41.65)	94.5 (53.89)	0.651
**Leptin (ng/mL)**	33.52 (13.11)	27.54 (7.83)	0.320
**MVPA (min/day)**	2.6 (1.14)	2.21 (1.08)	0.486
**CEBQ**	1.00(0.12)	1.19 (0.44)	0.356

Data are mean (SD), p[1] Unpaired T –Test for the comparison between the two groups.

Supplementary Table 3. Changes in anthropometric and biochemical from a subpopulation of matched age and sex subjects with the Ile251Leu MC4R mutations and without the mutation.

8-wek<br>1 year	Ile251Leu MC4R mutation<br>N=5<br>N=4	No MC4R mutation<br>N=21<br>N=13	p[1]
Δ Height (cm)			
8-wek	1.42 (0.46)**	1.13 (0.50)***	0.257
1 year	6.44 (2.80)**	5.82 (0.63)***	0.437
Δ Weight (Kg)			
8-wek	-2.54 (2.54)	-2.66 (2.14)***	0.909
1 year	2.47 (6.52)	1.73 (6.44)	0.843
Δ BMI (Kg/m^2)			
8-wek	-1.8 (1.29)**	-1.68 (1.06)***	0.837
1 year	-1.2 (3.14)	-1.19 (3.18)	0.996
Δ BMI-SDS			
8-wek	-0.74 (0.41)*	-0.57 (0.479)***	0.477
1 year	-1.02 (1.21)	-0.69(0.85)*	0.523
Δ WHR			
8-wek	-0.02 (0.01)*	-0.03 (0.02)***	0.234
1 year	-0.04 (0.03)	-0.02 (0.02)***	0.163
Δ Glucose (mg/dL)			
8-wek	2.33 (5.68)	-2.47 (6.67)	0.258
1 year	6.01 (0)	-1.54 (7.77)	0.214
Δ Insulin (μU/mL)			
8-wek	3.16	-0.57 (4.48)	0.266
1 year	6.80 (1.27)	-3.34 (10.89)	0.234
Δ HOMA-IR			
8-wek	0.71 (1.71)	-0.16 (1.02)	0.245
1 year	1.65 (0.22)	-0.82 (2.68)	0.238
Δ Total cholesterol(mg/dL)			
8-wek	-29 (11.53)*	-15.05 (24.97)*	0.363
1 year	25.00 (1.41)*	7.81 (26.51)	0.832
Δ HDL-cholesterol (mg/dL)			
8-wek	-3.66 (6.65)	-5.05 (7.85)	0.776
1 year	-5 (1.41)	0.44(5.15)	0.187
Δ LDL-cholesterol (mg/dL)			
8-wek	-24 (4.35)**	-7.4 (18.51)	0.148
1 year	-21 (5.65)	-2.92 (25.81)	0.373
ΔTriglycerides (mg/dL)			
8-wek	-6.33 (10.01)	-8.05 (35.83)*	0.963
1 year	6 (11.31)	-26.63 (54.89)	0.435
Δ Leptin (ng/mL)			
8-wek	-2.43 (15.85)	-11.57 (10.42)*	0.289
1 year	19.15 (16.47)	0.42 (17.91)	0.262

Data are mean (SD). Paired T-test for changes between baseline vs 8 week, and baseline vs. 1 year (* <0.05, ** <0.010,***<0.001); p[1] Unpaired T –Test for the comparison between the two groups.
